# Supplementary material for: Barriers and facilitators to oral pre-exposure prophylaxis uptake among adolescents girls and young women at elevated risk of HIV acquisition in Lilongwe, Malawi: A qualitative study
Source: PLOS Glob Public Health. 2025 Apr 7;5(4):e0004006. doi: 10.1371/journal.pgph.0004006 (PMC11975068; doi:10.1371/journal.pgph.0004006)
Supplement: S1 Appendix — (DOCX) [file pgph.0004006.s001.docx]

**S1 Appendix:** **Interview guide for AGYW-English version**

**PARTICIPANT ID: ___________**

**DATE OF INTERVIEW: ____________ / ________/ ____________/**

**Section A- INTRODUCTION**

Thank you so much for agreeing to have this interview with me. Before we start, I

would like to know more about you.

**Section B DEMOGRAPHIC DATA**

Date of birth: ____/____/_____ (DD/MM/YYYY)

| **Age** | **Marital status** | **Number of Children** | **Education Status** | **Occupation** | **Religion** | **PrEP Status** |
| --- | --- | --- | --- | --- | --- | --- |
|  |  |  |  |  |  |  |

**Section C - Perceptions on HIV and PrEP Services:**

**Vignette**

***Interviewer: I’d like to tell you a story about a teenage girl called Maria and her experience with sexual life. I will tell you part of the story and would like you to assist me in completing it.***

Maria, a 17-year-old girl, wants to use oral PrEP but is too scared to tell her boyfriend, Victor, as she fears he will react negatively. She is also nervous about her parents finding out, as she still lives at home. She strongly suspects that Victor has other partners. Victor is much older than Maria. He is 35 years old and gives her money for clothes and airtime. He monitors her closely and gets angry when she hangs out with other men or goes somewhere without telling him.

1. What is your understanding of PrEP services?

Probe on:

- Benefits of PrEP services amongst the AGYWs
- Concerns about PrEP use in AGYW (Probe on safety, efficacy, side effects**)**

**Section D- Barriers to uptake and utilisation of PrEP**

1. Please explain to me in detail the factors that impede Maria's uptake and utilisation of PrEP services.
2. Explain to me in detail the factors that impede the uptake and utilisation of PrEP services by You. Probe on:

- Knowledge of PrEP
- Stigma and discrimination (partner, family, community and peers)
- PrEP Experience (PrEP side effects)
- Quality of health services offered at the facility
- Healthcare worker attitude towards AGYW on the use of PrEP
- Distance travelled to the health facility
- Operation hours of the facility.

**Section D- Enablers to PrEP services uptake and utilisation**

1. Explain the factors enabling Maria to access and use PrEP services.
2. Please explain the factors enabling you to access and use PrEP services.

Probe on

- Knowledge of PrEP
- Encouragement by relationships (partner, family, community and peers)
- Quality of health services provided at the facility
- Distance to the health facility
- Healthcare worker attitude towards AGYW on PrEP
- Operation hours of the facility
