# Supplementary material for: Barriers and facilitators to oral pre-exposure prophylaxis uptake among adolescents girls and young women at elevated risk of HIV acquisition in Lilongwe, Malawi: A qualitative study
Source: PLOS Glob Public Health. 2025 Apr 7;5(4):e0004006. doi: 10.1371/journal.pgph.0004006 (PMC11975068; doi:10.1371/journal.pgph.0004006)
Supplement: S2 Appendix — (DOCX) [file pgph.0004006.s002.docx]

**S2 Appendix: Interview Guide for Health Care Workers in English**

**Age:**

**Gender:**

**Cadre:**

**Years of Service:**

**Section A -Welcome Remarks**

1. Tell me about PrEP.
2. What is your role at this facility?
3. What is your role in PrEP Services?

**Section B - Perceptions on PrEP Services among AGYW:**

1. What is your understanding of PrEP services among AGYW:

- What are the benefits of PrEP services amongst the AGYWs
- What concerns, if any, do you have about PrEP use in AGYW? (Probe on safety, efficacy, drug resistance, risk compensation**)**

**Section C- Barriers to PrEP**

1. Explain in detail the factors that impede the provision of Pre-Exposure prophylaxis services at this facility.

Probe on:

- Availability of resources at the facility.
- Volume of Work to be attended per HCW.
- If the HCWs are well trained about PrEP among AGYW
- Capabilities and Knowledge
- If culture affects the work of PrEP uptake
- Operation hours of the facility in providing PrEP
- The flexibility of operating procedures such as screening procedures
- Legal and ethical considerations when handling adolescents

1. Please explain to me the client factors that you think may impede the uptake of Pre-Exposure Prophylaxis services in this area. Probe on:

- PrEP knowledge, attitudes and perceptions
- Stigma and discrimination
- Relationships (partners, parents, peers, community)
- Clinic Experience
- PrEP side effects

**Section D- Facilitators to PrEP Services**

1. Please explain the factors enabling healthcare workers to provide PrEP services at this facility. Probe on

- Availability of resources at the facility.
- Volume of Work to be attended per HCW.
- If the HCWs are well trained about PrEP among AGYW
- If culture affects the work of PrEP uptake
- Operation hours of the facility in providing PrEP
- The flexibility of operating procedures such as screening procedures
- Legal and ethical considerations when handling adolescents

1. Please explain to me the factors that enable AGYWs to take up PrEP services at this facility. Probe on:

- Community Norms (Eg. Religion)
- Education Status
- Occupation
- Knowledge of PrEP
- Relationships (partner, family, community and peers)
- Quality of health services
- Distance to the health facility
- Healthcare worker attitude
- Operation hours

**Section E- Closing Remarks**

1. We are now at the end of the Interview. Is there anything you would like to add concerning the provision and uptake of Pre-Exposure Prophylaxis services among Adolescent Girls and Young Women?

Thank you very much for your time.
