# Supplementary material for: Barriers and facilitators to oral pre-exposure prophylaxis uptake among adolescents girls and young women at elevated risk of HIV acquisition in Lilongwe, Malawi: A qualitative study
Source: PLOS Glob Public Health. 2025 Apr 7;5(4):e0004006. doi: 10.1371/journal.pgph.0004006 (PMC11975068; doi:10.1371/journal.pgph.0004006)
Supplement: Attachment — Submitted filename: Reviewers comments_Barriers and facilitators to Oral PrEP.docx [file pgph.0004006.s003.docx]

**Reviewer’s feedback:** Barriers and facilitators to Oral Pre-Exposure Prophylaxis Uptake among Adolescents girls and young women at elevated risk of HIV acquisition in Lilongwe, Malawi: A qualitative study.

**METHODS**

**Study setting**

STI cannot be described as a service. Services may be either prevention of STI or treatment of STI or both. Authors should correct this.

Authors also did not mention maybe the health facility offers provision of PrEP as one of the services.

**Sampling and recruitment of study participants**

Authors may need to clarify if the 10 AGYW not on PrEP selected for the survey were not eligible for PrEP, or eligible and not offered or were offered but declined. This may be helpful to know as it may influence their responses.

Were the 10 HCWs selected the ones trained on PrEP or a mixture of those trained and those not trained?

**Data collection**

Authors may need to clarify how the interviews were carried out……the interview of the AGYW seems like a form of exit interview, that is they were interviewed after accessing services and leaving the facility. The interview was said to be carried out within 28 days, so how many respondents were interviewed per day, did all the required number of respondents (20) show up within that 28 days for services, or some needed to be called to come for the interview? Maybe additional information about AGYW client volume in Kawale health center and the AGYW PrEP clients’ visit/appointment frequency may be helpful to understand how the 20 respondents were reached in 28 days via exit interview method.

Another important part that may require clarity are the data collection instruments used, it was indicated that the interview guides and vignettes are attached as appendices but they were not seen. An in-depth interview in a qualitative survey is meant to explore respondents’ insight as much as possible, so the instrument used may be better if not structured.

**RESULTS**

Beyond the feedback from the HCWs, it may be nice to get/document the perspective of the AGYW concerning the attitude of HCWs, and the issue of lack of privacy in the facility. The perspective of the AGYW with regards to these factors will be very helpful to guide our recommendation because they are the recipients of the services provided by the HCWs and the facility.

The perspective of the HCWs concerning the issue of stigma (outer setting factor) may also be documented from this survey if available. As mentioned in the discussion section where some HCWs are reluctant in offering PrEP to AGWYs because of the concern of increase inappropriate sexual behavior and possible ARV drug resistance among the AGYW, are there such among the HCWs interviewed in the Malawi study too, especially if such HCW has not been trained on PrEP provision?
