# Supplementary material for: Barriers and facilitators to oral pre-exposure prophylaxis uptake among adolescents girls and young women at elevated risk of HIV acquisition in Lilongwe, Malawi: A qualitative study
Source: PLOS Glob Public Health. 2025 Apr 7;5(4):e0004006. doi: 10.1371/journal.pgph.0004006 (PMC11975068; doi:10.1371/journal.pgph.0004006)
Supplement: Attachment — Submitted filename: Response to reviewers.doc [file pgph.0004006.s005.doc]

**Dear Reviewers and Editors**

We thank you for the review over our manuscript which has improved it. Please find below the responses to the queries raised.

**Reviewer #1**:

The authors explored the factors that facilitate PrEP uptake and barriers to the same among adolescent girls and young women at increased risk of acquiring HIV infections. I believe this is a significant study due to the low uptake of PrEP despite the demonstrated efficacy of the ARVs in preventing HIV infection.
Strengths: Including participants not on PrEP in the study provided an avenue to explore barriers affecting PrEP uptake.

Thank you for your observation and encouragement.

**Major Issues**
1.Introduction: reference number 16 which should have provided details on the targets for PrEP was not found. Authors should investigate that.

We have investigated the problem and resorted to delete the sentence that had a reference number 16 on page 4 as the reference webpage currently can not be found.

Authors should provide additional details on the PrEP cascade including how many AGYW that received HIV testing services tested negative, the number offered PrEP, and the number that accepted PrEP and enrolled. “This study deliberately chose non-probability sampling [19] to include both AGYW who accepted PrEP and those that were not on PrEP (10 AGYW in each group)”

All the 20 AGYW participants enrolled were tested for HIV and tested Negative. All participants were offered PrEP but 10 accepted and 10 participants declined.

2.Sampling: Mention was made of appendices 1 & 2 which contain the interview guides and vignettes but are not included in the paper. Is this a deliberate omission?

Thank you for noting this. The appendices have been included in the paper

3.Results: Authors to clarify if side effects were barriers to PrEP uptake or PrEP continuation. Is it fear of side effects or presence of side effects?

The side effects were mentioned as barriers by both AGYW that are on PrEP and those that are not on PrEP. Hence the side effects were barriers to PrEP uptake and PrEP continuation and this has been corrected in the paper on page 15 and 16.

**Minor Issues**
1.Sampling: Study Authors should clarify the categories of participants recruited into the study. Those not on PrEP, were they offered PrEP and found to be eligible for PrEP but declined?

The 10 AGYW were those who were eligible but declined PrEP due to various reasons which have been included in the results and discussion.

**Reviewer #2:**

Thanks very much to the authors for the opportunity to read this manuscript. Overall, the topic is important, and with greater detail in the results section and improved connection between the framework used to design the study and the discussion section, it will be a nice contribution to the literature.

I would recommend a review for clarity of language and grammatical issues throughout prior to resubmission.

The paper has been reviewed for clarity of issues and grammatical issues has been addressed.

**Abstract:**

In the results section of the abstract, there are a lot of different themes summarized in two long sentences. It would help if the themes for AGYW were discussed in separate sentences from those for HCW and more detail included. For example, it is said that HCW attitudes (there's a typo - it says altitude but that should read attitudes) are facilitators, but I could see those potentially being barriers depending on what the attitude specifically is. More detail should be included to clarify.

The sentences have been revised. The themes for AGYW and HCW has been separated into different sentence in the results section on page 2.

In the conclusion of the abstract, the statements made are not clearly built upon the results highlighted in the previous section. For example, what is the comprehensive strategy that is needed that is tailored?

The statement has been revised to clearly build upon the results in the results section on page 3.
 **Introduction:**

**Paragraph 2:** In the first line it says that the disparities in the impact of HIV are attributed to high risk behavior. Do you mean among AG YW or more broadly among youth?

The statement “The disparities in the impact of HIV are attributed to high risk behavior”, applies only to AGYW and it has been clarified on page 3 and 4.

Space in addition you say that place of residence has also been associated with risk of HIV. Clarifying which places have an increased risk would be helpful for readers unfamiliar with this topic.

We have added information that is explaining more on the residence that are associated with risk of HIV in the introduction section paragraph 2 on page 4.

In the sentence that begins with “despite availability of female condoms...,” there is a typo with the word usage. Should that be use?

The word use has been revised to “usage” on page 12.

In addition, would it be helpful here to explain the reason why women have limited ability to use or negotiate economies? For example, gender inequitable norms that influence dynamics?

New information has been added to explain the reason why women have limited ability to negotiate condoms on page 4.

I would also recommend starting a new paragraph before the sentence that begins “another HIV prevention intervention is...”

The paragraph has been revised into two paragraphs on page 4.

**Paragraph 3:** if there has been limited research detailing the factors that influence PrEP uptake, what has that limited research shown? If there has been no research to date, then it should be explicitly stated.

The sentence has been revised to state that there has been no research to date detailing the factors that influence PrEP uptake on page 4.

Since the study setting is an urban area it might be helpful to spotlight findings of prep uptake in urban versus rural areas. Is it higher in urban areas in Malawi or not, for example? That would help support your study justification a bit more.

This study was conducted in an urban setting and we didn’t compare with any other rural setting. Currently in Malawi, PrEP implementation has been focusing in the urban areas where most of the key populations are found. We recommend another study that can look into the differences in PrEP uptake between urban and rural health facility. This would benefit from the programmatic data on the uptake of PrEP in Lilongwe urban

**Methods:**

Study setting: in the sentence starting “The services offered…,” there is a missing “are” after the word include.

The sentence has been revised to include “are” under study setting on page 5.

**Sampling:** while you used purposive sampling, it would be helpful to give more details about how you recruited participants. For example, did it take place in person, using flyers, or another method?

We have provided the details on how the participants were recruited on paragraph 1 on page 6 as follows:

Participants were approached at the facility by the investigators and the PrEP focal person of the facility and were briefed of the study. They were interviewed at their convenience upon agreement to participate in the study.

**Data collection:** What do you mean by “well-trained?” what training was provided?

The female research assistant was well trained in our protocol and also has a background of qualitative study data collection training.

Why was the CFIR framework used to guide the interview guide? How? Can you elaborate a bit more?

The CFIR provides a menu of constructs arranged across 5 domains that can be used in a range of applications. It is a practical framework to help guide systematic assessment of potential barriers and facilitators in implementation research. It is used to identify factors that influence intervention implementation and effectiveness. So the questions for the interview guide were formed with reference to the CFIR constructs from the main domains.

**Data analysis**: Change second sentence to “Data “were” managed…” In addition, the authors jump from familiarization with transcripts to talking about how codes are compared, but there should be a bit more discussion of how the codebook was developed and tested. How can codes be compared before developing the coding framework? How did the authors use the CFIR framework to guide coding? Once coding was complete, how did the authors identify salient themes? What process was used?

The paragraph has been revised and now reads as follows:

The transcripts were analysed using a thematic analysis approach as suggested by Braun and Clarke [22]. Data were managed using qualitative software NVivo.

SN and ALNM read initial transcripts independently, repeatedly, to familiarize themselves with the data and at the third round of reading they independently coded the transcripts to develop a code book. The coding process followed deductive and inductive approaches. We use the CFIR framework to deduce the codes while the inductive codes were realised from the data. After independently coding the data, SN and ALNM discussed the initial codes that were developed and discussed areas where they differed until resolution. SN developed the entire codebook that was checked by ALNM and by AKB. SN then coded the entire dataset using the codebook while also adding more inductive codes as realised from the data. transcripts several times while listening to the audios in order to become completely immersed in the facts and guarantee proper transcription. Any interesting ideas and patterns the data may reveal will also be noted by the researchers. At the time of coding SN had periodic meetings with ALNM and AKB to oversee the progress and discuss other emerging issues or codes. After all the transcripts were coded, we combined related and comparable codes under an overarching theme that were either deduced from the conceptual framework or realised from the data. We reviewed the themes realised multiple times to ensure that they are representative of the data and were coherent. The matrix that compared the conceptual framework and the themes realised is shown in Table 1. The quotes attached to the themes were thoroughly reviewed to ensure that they were representative and that there was no overlap. The final themes were then reviewed by all researchers to ensure that they were correct and complete.

Refer to page 7 and 8

**Results:**

In the demographic characteristics section, it might be helpful to clarify what the different HCWs’ roles are in particular related to PrEP administration.

The HCWs’ roles has been included in the demographic characteristics section on page 8

In table 1 under Characteristics of Individuals, Facilitators – the summary is described as “knowledge of PrEP,” but what is written extends beyond PrEP to include knowledge of HIV risk susceptibility as well.

The extension that included HIV risk susceptibility has been removed in table 1 under results on page 9.

In the Inner Setting section, under burden of work, it would be helpful to have the authors elaborate more on why high workload was considered a barrier to PrEP uptake.

We have provided more information about high workload under the inner section on page 16

In addition, some of the factors mentioned seem like they might fit better in the “outer setting” section. Perhaps why the authors have bucketed them in “inner setting” could be clarified.

The inner setting is the setting in which the innovation is implemented, e.g., hospital, school, city. There may be multiple Inner Settings and/or multiple levels within the Inner Setting, e.g., unit, classroom, team. The inner setting in this study meant the aspects or factors that take place within the facility that are not influenced by outer forces. In this case those factors apply.

In the outer setting section, the patient needs and resources under facilitators is not clear.

The sentence in the outer setting section about patient needs and resources under facilitator has been revised and make it clear on page 10.

**Facilitators: knowledge** – the first sentence is quite long, with some grammatical issues, and should be shortened and made into multiple sentences to explain the findings in greater depth.

The sentence has been shortened into small sentences under facilitator knowledge on page 11

In the translated quotes throughout the results, I would suggest minor tweaks to the grammar to retain the original meaning but make them more easily understood by the reader.

The translated quotes has been reviewed and minor tweaks to the grammar have been made.

**Facilitators: inner setting** – the availability of resources section is quite short and could have greater detail.

“ The availability of resources” section has been revised to give a greater detail on page 14. It now reads as follows: HCWs argued that the availability of PrEP, as well as other resources such as HIV test kits, makes it easier for AGYWs to receive PrEP and benefit from it. Participants were encouraged with the availability of PrEP drugs at the facility and they knew that the drugs are available at all times.

In addition, I wonder if it makes sense for this to be in the “inner setting” section or the outer setting?

Thank you for that observation. In this study, the issue to do with resources are being discussed at a facility level. For example, the problem may be lack of ordering of drugs and other resources at the institution and not that the resources are out of stock at national level.

Barriers: Adherence – The point about abuse of PrEP could be clarified further to illustrate the point being made.

The point about abuse of PrEP has been clarified on page 15 and it now reads as follows:

The side effects of PrEP medications remain a concern among PrEP beneficiaries. HCWs and AGYW reported that AGYW fear PrEP side effects hence they may not adhere to its usage. Others were concerned that PrEP could lead to drug resistance or promote risky behaviours among AGYW as they believed PrEP was not 100% effective. Abuse of PrEP was also highlighted that some AGYW would come to the clinic to get PrEP not because they wanted to use it but as a peer pressure. In addition, AGYW may abuse PrEP by sharing with friends without prescription leading to inadequate dosing.

**Barriers: workload** – It would be helpful to expand the theme around workload to clarify why exactly this is a barrier for PrEP uptake.

The theme “work load” has been clarified on page 16 and it now reads as:

HCWs noted that workload on the side of providers and shortage of qualified PrEP providers hindered quality PrEP provision to the AGYW. It was noted that the clinician who attended to the clients at the OPD was also responsible for the dispensation of PrEP. The participants reiterated that in an environment that already suffers from staff shortages, the incorporation of PrEP as an additional clinic service was a challenge. They stated that the providers are already responsible for OPD services such that adding the role of being PrEP providers increases their scope of work.

Similarly, when talking about limited resources, it is mentioned that they are unable to conduct some of the critical tests. Can the authors incorporate an additional discussion around how this then prevents people from using PrEP? The point is implied, but I think it can be made clearer by the authors.

The addition information about limited resources has been added to clarify the point on page 18.
 **Barriers: outer setting** – It would be nice to incorporate more detailed discussion of the theme around stigma, as this is an important emergent finding to highlight, particularly in terms of programs working to improve uptake.
Thank you for that observation. We have incorporated more detailed discussion of the them around stigma on page 19.

Some HCWs explained that despite having most resources available, they do not conduct some critical tests at the facility due to lack of resources. They gave an example of a creatinine clearance test which is one of the indicators of PrEP eligibility. The HCWs reported that the only way to get the test done was to refer the patient to a tertiary hospital which was not always feasible. The participants stated that clients needed to receive PrEP services at a single facility than being referred to other facilities for other tests which becomes a deterrent.

**Discussion:**

It is important that the authors highlighted some of the findings around those with sex work experience. How many participants had this experience? How did the authors ensure saturation was reached with that theme as it was not part of the sampling strategy?

Its true that this theme was not part of sampling strategy only that among the AGYW that gets PrEP at Kawale health facility, some had sex work experience. Further discussion has been included as follows;

“Among the AGYW participants that were using PrEP, six had sex work experience that made them vulnerable to HIV infection. We noted that the participants who were on PrEP were encouraged by their exposure to HIV than those who were not on PrEP.”

Paragraph beginning with “In order to…” – suggest removing “really” in the first sentence to make the writing more formal.

The word “really” has been removed.

In addition, I am not sure I understand how visiting youth friendly services improves social capital. This point would be stronger if elaborated on in greater depth.
The point about “ visiting youth friendly services improves social capital” has been elaborated on page 24.

Paragraph beginning “We discovered…” – It would be helpful to repeat the specific demographic groupings here to inform policy and program thinking.

The demographic groupings have been included in the paragraph beginning “we discovered” under discussion section on page 21.

It would be helpful to explain the implications of the point around stigma and perceived risk compensation made in the discussion. Do the authors see these as connected? What are the programmatic and policy implications of this? How does this link back to the findings in the results?

Thank you for this observation. These two are different. The part that explains more on provider stigma and risk compensation are not the finding of this study. This information is from another study in the literature. In order to make it well understood, we have decided to remove that part of the paragraph from the paper under discussion section on page 22.

In addition, it might be helpful to circle back to the CFIR framework in the discussion, given its important role in the study design and analysis. What are the implications of these findings informed by the CFIR?

The discussion about the framework has been included under discussion and it reads as follows on page 25;

Our use of CFIR in this work has shown that we understand the challenges, facilitators, and contextual elements that are specific to PrEP implementation. According to what we've learned, specific recommendations for utilizing the CFIR include applying it in line with the research's implementation phase and integrating it into study design, data gathering, and analysis. An organized, thorough, and prompt knowledge of the obstacles and enablers to PrEP uptake was facilitated by using the CFIR to direct data collection and reporting of findings.

**Reviewer’s 3 3**

**METHODS**

**Study setting**

STI cannot be described as a service. Services may be either prevention of STI or treatment of STI or both. Authors should correct this.

We have revised this sentence to STI treatment and prevention. Please see page 5, paragraph 1 under study setting.

Authors also did not mention maybe the health facility offers provision of PrEP as one of the services.

We have included HIV PrEP as one of the services too. Please see page 5, paragraph 1 under study setting.

**Sampling and recruitment of study participants**

Authors may need to clarify if the 10 AGYW not on PrEP selected for the survey were not eligible for PrEP, or eligible and not offered or were offered but declined. This may be helpful to know as it may influence their responses.

The 10 AGYW were those who were eligible but declined PrEP due to various reasons which have been included in the results and discussion.

Were the 10 HCWs selected the ones trained on PrEP or a mixture of those trained and those not trained?

All the 10 HCWs were trained on PrEP.

**Data collection**

Authors may need to clarify how the interviews were carried out……the interview of the AGYW seems like a form of exit interview, that is they were interviewed after accessing services and leaving the facility. The interview was said to be carried out within 28 days, so how many respondents were interviewed per day, did all the required number of respondents (20) show up within that 28 days for services, or some needed to be called to come for the interview?

We were having a minimum of 2 interviews per day and a maximum of 3 interviews. Some AGYW were interviewed after their clinic visit while others were called and asked if they were interested in the study by the facility’s PrEP focal person.

Maybe additional information about AGYW client volume in Kawale health center and the AGYW PrEP clients’ visit/appointment frequency may be helpful to understand how the 20 respondents were reached in 28 days via exit interview method.

The facility had enrolled 140 women on PrEP around the implementation period. Of the women initiated on PrEP, 28 were adolescents and young women, representing 20%.

Another important part that may require clarity are the data collection instruments used, it was indicated that the interview guides and vignettes are attached as appendices but they were not seen. An in-depth interview in a qualitative survey is meant to explore respondents’ insight as much as possible, so the instrument used may be better if not structured.

The HCWs IDI guide had open-ended questions and not structured questions but the vignette had structured questions. We have made the changes under data collection on page 6 and 7. The data collection instruments have been attached to the paper as appendices.

**RESULTS**

Beyond the feedback from the HCWs, it may be nice to get/document the perspective of the AGYW concerning the attitude of HCWs, and the issue of lack of privacy in the facility. The perspective of the AGYW with regards to these factors will be very helpful to guide our recommendation because they are the recipients of the services provided by the HCWs and the facility.

We have included the perspectives of the AGYW concerning the altitude of HCWs under the results section on page 22.

The perspective of the HCWs concerning the issue of stigma (outer setting factor) may also be documented from this survey if available. As mentioned in the discussion section where some HCWs are reluctant in offering PrEP to AGWYs because of the concern of increase inappropriate sexual behavior and possible ARV drug resistance among the AGYW, are there such among the HCWs interviewed in the Malawi study too, especially if such HCW has not been trained on PrEP provision?

The point about the HCWs concerning the issue of stigma was not reported in our study but in other studies. Therefore, that paragraph has been removed from the paper in order to be specific with our findings.

We look forward to your review,

Simon Nicholas (Corresponding Author)
